# Cross-Sectional Analysis of Sugar-Sweetened Beverage Consumption, Food Security, and Nativity Among Adults: Associations from National Health and Nutrition Examination Survey 2007–2020

**DOI:** 10.3390/nu17030520

**Published:** 2025-01-30

**Authors:** Aikaterini Stamou, Karen R. Flórez

**Affiliations:** Environmental, Occupational and Geospatial Sciences Department, Graduate School of Public Health and Heath Policy, City University of New York, New York, NY 10017, USA; aikaterini.stamou04@sphmail.cuny.edu

**Keywords:** acculturation, food insecurity, ultra-processed foods, public health nutrition, beverage consumption patterns

## Abstract

Background/Aims: Immigrant populations face unique dietary challenges influenced by food security and acculturation, particularly regarding sugar-sweetened beverages (SSBs). This study examines the interplay of nativity and food security on SSB consumption patterns. Methods: Data from the National Health and Nutrition Examination Survey (NHANES) 2007–2020 (N = 23,331) were analyzed in this cross-sectional study. SSB consumption was assessed through 24 h dietary recalls. Food security was categorized as high/marginal or low/very low, and nativity as U.S.-born or foreign-born. Stratified regression models by sex evaluated associations between nativity, food security, and SSB consumption. Results: U.S.-born adults with low food security had the highest sugar intake (men: β = 27.5, 95% CI [14.8, 40.3]; women: β = 30.4, 95% CI [11.0, 49.7]) and SSB consumption (men: β = 14.7, 95% CI [11.2, 18.3]; women: β = 23.6, 95% CI [15.2, 31.9]). Conclusions: The findings highlight associations that suggest the importance of culturally tailored interventions targeting vulnerable groups to address disparities in SSB consumption influenced by food security and nativity.

## 1. Introduction

The United States faces significant public health challenges with concurrent issues of low food security and obesity. Low food security, defined by the USDA as limited or uncertain access to adequate food, affects approximately 11% of U.S. households. Recent studies indicate that food insecurity is associated with a higher intake of ultra-processed foods, which is linked to obesity and cardiometabolic diseases [1]. Simultaneously, the prevalence of obesity is notably higher among those experiencing low food security, reflecting a paradox that underscores the need for further exploration of the underlying associations. Moreover, the role of ultra-processed food is critical to this problem, with more than half of U.S. adults consuming at least one sugar-sweetened beverage (SSB) daily [2].

Dietary choices such as the consumption of SSBs are also shaped by powerful cultural norms. Dietary acculturation, or the process by which immigrants begin to adopt the dietary patterns of the majority, is of particular importance for immigrant groups in the U.S. [3]. Low food security has been linked to a higher intake of ultra-processed foods, which is associated with obesity and other adverse health outcomes [1]. The literature also suggests that participation in food assistance programs such as the Supplemental Nutrition Assistance Program (SNAP) can modify the impact of food insecurity on dietary choices with varying effects on diet quality [4,5].

The consumption of ultra-processed foods and SSBs is particularly high among low-income and food-insecure populations, who often participate in programs such as SNAP [1]. The adoption of Western dietary patterns, characterized by increased intake of processed foods and sugar-sweetened beverages, has been linked to escalating obesity rates within these communities [6,7]. Higher levels of ultra-processed food consumption are observed among individuals experiencing severe food insecurity, suggesting a strong correlation between food insecurity and poor dietary quality [3].

SSBs, in particular, are of significant concern because of their high calorie content but low nutrient content, which contributes to obesity, type 2 diabetes, cardiovascular diseases, and adverse dental health outcomes [8,9]. The transition to a diet rich in SSBs among various immigrant populations reflects broader socioeconomic and environmental factors, including limited access to affordable and nutritious food and the aggressive marketing of unhealthy food options [10]. Additionally, studies have shown that food insecurity is inversely associated with diet quality, further exacerbating health disparities [11,12].

The influence of cultural assimilation on dietary behavior, especially SSB consumption, is well documented, with studies indicating a direct correlation between the level of acculturation and SSB intake among U.S. immigrants [13,14]. This body of research suggests that the degree of cultural adaptation significantly affects dietary choices, which in turn influences health outcomes. As immigrants acculturate, they often shed traditional dietary behaviors and adopt those prevalent in the U.S., leading to increased consumption of energy-dense and nutrient-poor foods [3]. For instance, highly acculturated Latino immigrants tend to adopt dietary patterns prevalent in the U.S., including a higher consumption of SSBs, which is associated with an increased risk of type 2 diabetes and obesity [10]. However, a notable gap exists in our understanding of how the interplay between food security and acculturation contributes to adults’ dietary choices.

This study aims to bridge this knowledge gap by examining the associations between food security, acculturation, and dietary choices among diverse immigrant populations in the United States, specifically focusing on SSB consumption as a dietary marker of acculturation. We hypothesize that U.S.-born adults with low food security will exhibit the highest levels of sugar and SSB consumption compared to their immigrant counterparts, consistent with the literature suggesting that acculturation is associated with lower dietary quality.

## 2. Materials and Methods

### 2.1. Study Design

**Figure 1:**
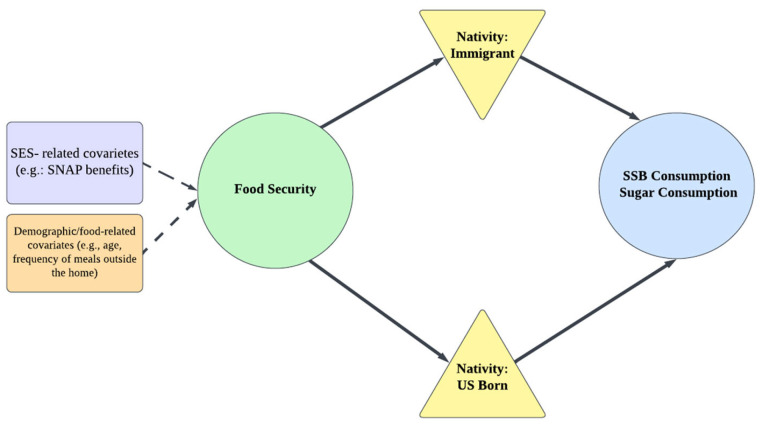
Conceptual model illustrating the interaction between food security and nativity on sugar-sweetened beverage (SSB) consumption.

This cross-sectional study used data from the National Health and Nutrition Examination Survey (NHANES) 2007–2020. NHANES provides nationally representative data on the health and nutritional status of the U.S. civilian, noninstitutionalized population. Six NHANES cycles (2007–2020) were analyzed to avoid potential dietary behavior changes during the COVID-19 pandemic. We developed a conceptual model to guide the selection of variables for the regression analysis (Figure 1). The model illustrates the associations between food security, nativity, and dietary outcomes, with covariates included to control for potential confounders. Variables with theoretical relevance and prior empirical support were prioritized for inclusion, while those with high collinearity or excessive missing data were excluded to ensure robust analyses. Figure 1 provides a visual summary of these interconnections.

#### Participants

**Figure 2:**
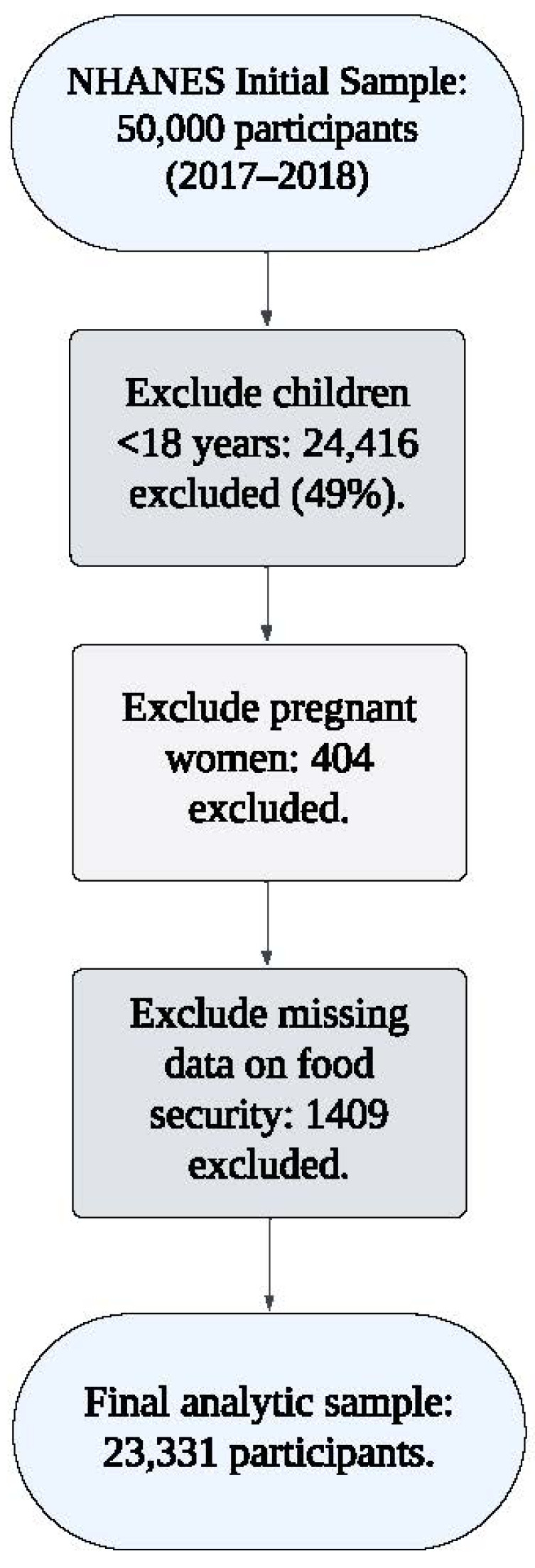
Flowchart of sample selection for the NHANES 2017–2018 analysis.

The analytic sample included 23,331 adults aged 18 and older. As shown in Figure 2, exclusions were made for pregnant women (n = 404), children under 18 years (n = 24,416), and individuals with missing data on adult food security status (n = 1409). All participants provided informed consent, and the study was conducted in accordance with the Declaration of Helsinki.

### 2.2. Outcomes

Sugar intake: The primary outcome of interest was sugar consumption, measured in grams (g). Data on the types and quantities of foods and beverages consumed were obtained through the NHANES dietary interview component, which utilizes a 24 h dietary recall conducted over two non-consecutive days. This method ensured an accurate assessment of sugar intake within the study population.

SSB consumption: The consumption of SSBs was quantified in grams of the total sugars derived from these beverages. Data on the types and quantities of foods and beverages consumed were obtained through the NHANES dietary interview component, which utilizes two non-consecutive 24 h dietary recalls. The SSB identification utilized 133 USDA food codes, covering a range of sweetened beverages, excluding alcoholic drinks.

### 2.3. Exposures

Household adult food security: Utilizing the US Household Food Security Survey Module developed by the USDA, adult food security status was assessed. NHANES categorizes these data into four levels: high, marginal, low, and very low. For analytical purposes, these were simplified into binary variables: ‘0’ for high/marginal food security and ‘1’ for low/deficient food security. Our study follows standard practices for distinguishing food-secure versus food-insecure households, as seen in prior research [5,15,16,17]. The USDA food security scale, which we employed, is a validated tool widely used to categorize households into food-secure or food-insecure groups. Dichotomizing food security allows for clearer comparisons across key variables, aligning with methodologies used in similar studies of dietary behaviors and health outcomes.

Nativity status: Nativity status was dichotomized to differentiate between U.S.-born individuals (coded 1) and those born outside the U.S. (coded 0). This distinction is crucial for analyzing dietary outcomes related to acculturation levels among Latinos and other ethnic groups [18,19,20]. Because we hypothesized a potential interactive effect between household food security status and nativity on sugar intake, we constructed a 4-level category variable indicating high or low food security for U.S.-born immigrants, which others have noted is better than relying only on multiplicative interaction measures (a statistical approach used to evaluate whether the combined effect of two variables on an outcome differs from the sum of their individual effects) [21]. This is consistent with previous studies that have tested the joint effect of food insecurity among vulnerable populations, including immigrants [4,22].

### 2.4. Covariates

Covariates included age (categorized as 18–29, 30–49, and 50–64 years), sex, race/ethnicity (Latino, non-Hispanic White, Black, Asian, and other/multiracial), educational attainment (less than high school, high school graduate, and more than high school), marital status, poverty index (categorized as <1.30, 1.30–1.85, and >1.85), SNAP benefits, and weekly frequency of meals not prepared at home.

### 2.5. Statistical Analysis

Weighted percentages and standard errors (SEs) were used to describe categorical variables, and the mean and SE were used to describe the distribution of continuous variables in descriptive statistics for men and women, given past research documenting differences in dietary intake by sex [23,24,25]. Preliminary analyses revealed significant interactions between key exposure variables and sex, justifying stratification. Stratified linear regression models were used to examine the association between household food security, nativity, and sugar consumption, including the percentage of sugar consumption from SSBs, among men and women. We fit unadjusted models, where the joint food security/nativity variable was the only predictor for men and women because this approach yielded the most public health relevance compared to the multiplicative interaction models [21]. Adjusted models accounted for the following covariates: age, sex, race/ethnicity, education level, marital status, and the Monthly Poverty Index. The Monthly Poverty Index, adjusted for family size and state, represents a household’s economic status categorized into three levels. Other food- or health-related covariates included receiving SNAP benefits (yes/no), number of meals prepared outside the home in a week, and weight categories. For all regression models, we used high food security/immigrant as the reference category because we hypothesized that it was the stratum with the lowest risk, which follows best practices [4] and previous research on immigrant populations [26]. A *p*-value of <0.05 was considered statistically significant, and all *p*-values were 2-sided.

### 2.6. Ethical Approval

The NHANES protocol was approved by the National Center for Health Statistics (NCHS) Research Ethics Review Board, and all participants provided informed consent.

## 3. Results

### 3.1. Participant Characteristics

Table 1 shows the descriptive statistics for the analytical sample of 23,331 adults from the NHANES data cycle from 2007 to 2020. Among the participants, 11,926 were male and 11,405 were female. Regarding household food security, 78.3% of the sample reported high food security, with a slightly higher prevalence among women (81.2%) than men (75.4%). The sample comprised 70% U.S.-born people and 30% immigrants, with women being more likely to be U.S.-born than men (74.6% vs. 68.4%). The age distribution showed a significant proportion of participants in the 30–49-year range (34.3%), followed by 50–64 years (26.1%). Notably, men had a higher representation in the 50–64-year range (27.3%) than women (24.8%). The educational level was high, with 61.9% having more than a high school diploma. Specifically, 64.1% of the men and 59.5% of the women had a high school diploma or higher. The race/ethnicity distribution indicated that 65.5% were non-Latino White, 14.6% were Latino, 11.1% were non-Latino Black, 5.7% were non-Latino Asian, and 3.4% were from other racial groups. The Latino subgroup included 14.0% men and 15.2% women. Marital status varied, with 55.6% married or living with a partner. Regarding the frequency of meals not prepared at home, 33.6% reported 3–6 meals per week, whereas 18.1% reported more than seven meals per week. The prevalence of obesity (BMI > 30) was 38.4%, with a slightly higher prevalence (39.8%) in men than in women (36.8%). The poverty index varied among the participants: 12.2% had a Monthly Poverty Index of less than 1.30, 25.1% had an index between 1.30 and 1.85, and 62.7% had an index greater than 1.85. Additionally, 30.3% of the sample reported having received SNAP benefits, with a slightly higher prevalence in men (32.4%) than in women (28.1%).

### 3.2. Sugar and SSB Consumption

Table 2 shows the mean sugar and SSB consumption stratified by food security and nativity status. U.S.-born participants with low food security reported the highest mean daily sugar intake and derived a larger proportion of their total sugar income from SSBs. For men, the mean sugar consumption was 173.3 g/day, with 35.9 g/day coming from SSBs. Among women, sugar consumption reached 219.4 g/day, with 60.4 g/day from SSBs.

### 3.3. Regression Analysis

Table 3 shows crude and adjusted regression models for sugar consumption. In the adjusted models, U.S.-born men and women with low food security had statistically significantly higher sugar consumption relative to their immigrant counterparts with high food security (men: β = 27.5, *p* ≤ 0.001; women: β = 30.4, *p* ≤ 0.001).

Table 4 presents the crude and adjusted regression models for the percentage of sugar consumption from SSBs. The results showed that U.S.-born individuals with low food security had the highest percentage of sugar intake from SSBs, with a more pronounced effect in women (men: β = 14.7, *p* = 0.001; women: β = 23.6, *p* = 0.001).

## 4. Discussion

Our study found that U.S.-born men and women with low food security have the highest sugar and SSB intake relative to their immigrant counterparts with high food security. This is consistent with past research that found higher acculturation is associated with lower dietary quality [12]. Specifically, among Mexican American adults in NHANES III, those born in Mexico were more likely to adhere to dietary guidelines [26], and recent findings suggest less healthy patterns also extend to individuals with diabetes [5,27]. Overall, evidence suggests that acculturation may exacerbate nutritional health disparities [28]. Acculturation in this study was approximated using nativity status (U.S.-born vs. foreign-born), a widely used proxy in public health research. While this analysis focused on nativity, the NHANES dataset also includes additional variables that could serve as proxies for acculturation, such as length of stay in the United States for foreign-born individuals and language preference (e.g., preferred interview language), which can provide further insights into acculturation. Although the dietary assimilation hypothesis has been supported in previous studies [10,12], the literature has primarily focused on fruits and vegetables and largely ignored the role of food security, except for a few notable studies among children [29]. We used nativity status as a proxy for acculturation in our analysis; however, future research should consider incorporating multidimensional measures of acculturation, such as length of stay in the United States and language use, to better capture its complexities. Our use of nationally representative nutritional data allowed us to investigate how food security and nativity are associated with dietary patterns, specifically sugar and SSB intake. This approach enabled us to comprehensively link food security status and nativity with dietary behaviors, providing insights into the intersection of these factors on nutritional disparities.

Specifically, previous research has shown that cultural adaptation is associated with increased consumption of fast food and SSBs and lower consumption of fruits and vegetables, indicating a shift toward a more Westernized diet [10,12]. Our findings further emphasize the importance of addressing food security and its interaction with acculturation to develop culturally tailored public health interventions. The literature thus highlights a need for subgroup analyses to better understand cultural influences on diet [13]. Although we did not find statistically significant results for immigrants with low food security in the fully adjusted models, bivariate associations suggested a trend. Longitudinal studies that incorporate robust measures of acculturation would help clarify these dynamics more fully, guiding culturally sensitive interventions [30]. In the results, the β-coefficients highlight notable differences in sugar and SSB consumption across food security and nativity groups. For instance, U.S.-born men with low food security consumed an additional 27.5 g of sugar per day compared to their immigrant counterparts with high food security. This represents a substantial dietary burden, equivalent to approximately seven teaspoons of sugar, exceeding recommended daily sugar intake limits set by dietary guidelines. Similarly, women in the same group consumed 30.4 g more sugar per day, with 23.6 g specifically from SSBs, reflecting a significant public health concern given the established link between SSB consumption and obesity, type 2 diabetes, and cardiovascular diseases.

These effect sizes demonstrate the meaningful differences in dietary patterns influenced by food security and nativity, emphasizing the urgent need for targeted interventions. Addressing these disparities could significantly reduce sugar intake and its associated health risks, particularly in vulnerable populations.

Low-income and minority groups are distinctly targeted by the beverage industry, which contributes to unhealthy food environments [31,32]. The low cost of high-calorie sweetened beverages, driven by agricultural subsidies, further establishes unhealthy dietary patterns [33]. Although our study draws from representative data, qualitative research such as Fielding-Singh’s “How the Other Half Eats” provides deeper insight into the lived experiences and negotiations immigrant families make around food, sometimes embracing Americanized foods for the joy and sense of belonging they confer [34]. Future research should explore these social and cultural dynamics further, particularly focusing on interventions that balance cultural preferences with healthy eating patterns. Nutrition educators should avoid assuming that immigrant families uniformly perceive American eating norms as unhealthy [35,36].

### Strengths and Limitations

Our study’s cross-sectional design limits causal inferences, and self-reported dietary data may introduce recall bias. Future work should consider multidimensional acculturation measures and explore differences among specific ethnic subgroups [37,38]. Recognizing these complex socio-cultural and economic dimensions is crucial for designing culturally sensitive, economically informed public health interventions that address broader health disparities.

## 5. Conclusions

In this study, we found significant associations between food security, nativity, and sugar and SSB consumption. U.S.-born individuals with low food security exhibited the highest levels of sugar and SSB intake compared to their immigrant counterparts with higher food security. These findings highlight the need for targeted public health interventions that address food security and cultural factors to mitigate dietary disparities and improve health outcomes. Future research should explore longitudinal approaches and include multidimensional measures of acculturation, such as language use and length of stay in the United States, to better understand the nuanced associations influencing dietary patterns.

To address these issues, public health policies should focus on expanding and tailoring food assistance programs, such as SNAP, to improve access to affordable, nutrient-dense foods for low-income U.S.-born individuals who are most at risk, as well as immigrant populations. Nutrition education programs are essential to promote healthier dietary behaviors among diverse communities. Stricter regulations on the marketing and availability of sugar-sweetened beverages are also needed, particularly in communities disproportionately targeted by the beverage industry. Furthermore, another policy tool is a SSB excise tax, as highlighted by the World Health Organization: “If governments tax products like sugary drinks, they can reduce suffering and save lives. They can also cut healthcare costs and increase revenues to invest in health services.”.

## Figures and Tables

**Table 1:** Descriptive characteristics for adults in NHANES ^a^ 2007 to 2020 by sex.

	Total Sample % N = 23,331	Men % n = 11,926	Women % n = 11,405
Household food security ^b^			
High	78.3	75.4	81.2
Low	21.7	20.2	23.4
Nativity			
United States	70	68.4	74.6
Other	28.7	26.3	30.2
Age, years			
18–29	20.7	19.5	21.9
30–49	34.3	33.6	35.1
50–64	26.1	26.4	25.9
≥65	18.9	20.6	17.1
Education			
<High School	12.9	12.0	13.7
High School	22.2	21.19	23.3
>High School	61.9	64.1	59.5
Race/Ethnicity ^c^			
Non-Latino White	65.5	65.3	65.7
Latino	14.6	14.0	15.2
Non-Latino Black	11.1	11.9	10.2
Non-Latino Asian	5.7	5.5	5.2
Non-Latino other	3.4	3.1	3.6
Marital status			
Married/living with partner	55.6	52.6	58.8
Single/widowed/divorced/separated	41.4	44.7	37.8
Number of meals not prepared at home (weekly)			
Never	16.6	18.3	14.6
1–2 meals/week	31.8	36.2	27.1
3–6 meals/week	33.6	32.5	34.7
7+ meals/week	18.1	13.0	23.4
Weight ^d^			
Underweight (<18.5)	1.6	2.1	1.2
Normal (18–25)	28.2	30.6	25.7
Overweight (>25–30)	31.8	27.6	36.3
Individuals with obesity (>30)	38.4	39.8	36.8
Monthly Poverty Index ^e^			
Index < 1.30	12.2	12.4	12.0
Index = 1.85	25.1	26.7	23.3
Index > 1.85	62.7	60.9	64.7
HH FS benefit: ever received			
No	69.7	67.6	71.9
Yes	30.3	32.4	28.1

^a^: Abbreviations: NHANES, National Health and Nutrition Examination Survey; ^b^: defined by the USDA as the measure of a household’s consistent access to adequate food for active, healthy living without disruption throughout the year; ^c^: self-identified race/ethnicity; ^d^: created using the measured body mass index and CDC classifications; ^e^: the Monthly Poverty Index represents the ratio of a family’s total monthly income to the poverty guidelines issued by the Department of Health and Human Services, adjusted for family size and state. A lower index indicates higher poverty, whereas a higher index indicates lower poverty.

**Table 2:** Mean sugar and SSB ^a^ consumption by nativity, food security and sex among adults in NHANES 2007 to 2020; N = 23,331.

	Low Food Security		High Food Security	
	Mean Sugar (SE) ^b^	Mean SSB (SE)	Mean Sugar (SE)	Mean SSB (SE)
Men				
US-born	173.3 (4.7)	35.9 (1.5)	161.2 (1.8)	22.2 (0.74)
Immigrant	142.7 (4.9)	22.9 (2.9)	128.6 (3.4)	14.5 (0.80)
Women				
US-born	219.4 (7.1)	60.4 (3.6)	197.9 (2.7)	33.1 (1.1)
Immigrant	167.3 (6.1)	36.6 (3.9)	160.9 (7.3)	26.6 (1.8)

^a^: sugar-sweetened beverages defined as beverages containing added sugars such as sucrose, high-fructose corn syrup, or fruit juice concentrates, which include sodas, fruit drinks, sports drinks, energy drinks, sweetened waters, and coffee and tea beverages with added sugars. ^b^: abbreviations: SE, standard error.

**Table 3:** Crude and adjusted models for the association between nativity, food security, and sugar consumption by sex among adults in NHANES ^a^ 2007 to 2020; N = 23,331.

	Men		Women	
	Unadjusted	Adjusted	Unadjusted	Adjusted
	**β ^b,c^ (95% CI)**	**β ^d^ (95% CI)**	**β ^b,c^ (95% CI)**	**β ^d^ (95% CI)**
Immigrant/High Food Security	1.00	1.00	1.00	1.00
Immigrant/Low Food Security	14.1 (2.9, 25.3)	6.9 (−4.9, 18.7)	6.5 (−8.5, 21.4)	−7.0 (−22.9, 8.9)
U.S-Born/High Food Security	32.6 (24.8, 40.5)	18.4 (9.7, 27.1)	36.9 (26.1, 47.8)	16.7 (2.5, 30.9)
U.S-Born/Low Food Security	44.7 (33.3, 56.1)	27.5 (14.8, 40.3)	58.6 (40.7, 76.4)	30.4 (11.0, 49.7)

^a^: abbreviations: CI, Confidence Interval; U.S., United States; NHANES, National Health and Nutrition Examination Survey, SNAP, Supplemental Nutrition Assistance Program; ^b^: boldface indicates statistical significance (*p* < 0.05); ^c^: beta estimates generated using STATA svy commands to account for complex sampling; ^d^: age (categorical), education (categorical), race/ethnicity (categorical), marital status (binary), weight (categorical), household poverty status (categorical), and SNAP benefits (binary) for adjusted model.

**Table 4:** Crude and adjusted models for the association between nativity, food security, and SSB consumption by sex among adults in NHANES ^a^ 2007 to 2020; N = 23,331.

	Men		Women	
	Unadjusted	Adjusted	Unadjusted	Adjusted
	**β ^b,c^ (95% CI) ^a^**	**β ^d^ (95% CI)**	**β ^b,c^ (95% CI)**	**β ^d^ (95% CI)**
Immigrant/High Food Security	1.00	1.00	1.00	1.00
Immigrant/Low Food Security	8.5 (4.8, 12.1)	3.0 (−0.52, 6.6)	9.9 (3.6, 16.3)	2.9 (−3.5, 9.2)
U.S-Born/High Food Security	7.8 (5.8, 9.7)	9.4 (7.3, 11.5)	6.4 (1.9, 10.9)	8.0 (3.1, 12.9)
U.S-Born/Low Food Security	21.5 (18.1, 24.8)	14.7 (11.2, 18.3)	33.8 (24.9, 42.6)	23.6 (15.2, 31.9)

^a^: abbreviations: CI, Confidence Interval; U.S., United States; ^b^: boldface indicates statistical significance (*p* < 0.05); ^c^: beta estimates generated using STATA svy commands to account for complex sampling; ^d^: age (categorical), education (categorical), race/ethnicity (categorical), marital status (binary), weight (categorical), household poverty status (categorical), and SNAP benefits (binary) for adjusted model.

## Data Availability

The dataset analyzed during the current study is publicly available at https://www.cdc.gov/nchs/nhanes/?CDC_AAref_Val=https://www.cdc.gov/nchs/nhanes/inde (available online 19 December 2024).

## References

[1] Leung C.W., Fulay A.P., Parnarouskis L., Martinez-Steele E., Gearhardt A.N., Wolfson J.A. (2022). Food insecurity and ultra-processed food consumption: The modifying role of participation in the Supplemental Nutrition Assistance Program (SNAP). Am. J. Clin. Nutr..

[2] Vega-López S., Armenta K., Eckert G., Maupomé G. (2020). Sugar-Related Dietary Behaviors and Dental Health Outcomes Among Hispanic Immigrants. Am. J. Prev. Med..

[3] Vatavuk-Serrati G., Kershaw K.N., Sotres-Alvarez D., Perreira K.M., Guadamuz J.S., Isasi C.R., Hirsch J.A., Van Horn L.V., Daviglus M.L., Albrecht S.S. (2023). Food Consumption Patterns and Diet Quality in Hispanic/Latino Immigrant Neighborhoods. J. Immigr. Minor Health.

[4] Lin Y.-C., Chang C.-S., Ho P.-S., Lee C.-H., Chen J.-H., Huang H.-L. (2019). Sugar-Sweetened Beverage Consumption and Early Childhood Caries: A Cross-Sectional Study of Taiwanese Children. J. Dent. Res..

[5] Flórez K.R., Albrecht S.S., Hwang N., Chambers E., Li Y., Gany F.M., Davila M. (2023). Household Food Security and Sugar-Sweetened Beverage Consumption Among Children in New York City. Public Health Nutr..

[6] Park S., Blanck H.M., Dooyema C.A., Ayala G.X. (2016). Association between sugar-sweetened beverage intake and proxies of acculturation among US Hispanic and non-Hispanic white adults. Am. J. Health Promot..

[7] Russo R.G., Northridge M.E., Wu B., Yi S.S. (2020). Characterizing Sugar-Sweetened Beverage Consumption for US Children and Adolescents by Race/Ethnicity. J. Racial Ethn. Health Disparities.

[8] Abraído-Lanza A.F., Echeverría S.E., Flórez K.R. (2016). Latino Immigrants, Acculturation, and Health: Promising New Directions in Research. Annu. Rev. Public Health.

[9] Abraído-Lanza A.F., Chao M.T., Flórez K.R. (2005). Do healthy behaviors decline with greater acculturation?. Soc. Sci. Med..

[10] Abraído-Lanza A.F., Armbrister A.N., Flórez K.R., Aguirre A.N. (2006). Toward a theory-driven model of acculturation in public health research. Am. J. Public Health.

[11] Popovic-Lipovac A., Strasser B. (2015). A Review on Changes in Food Habits Among Immigrant Women and Implications for Health. J. Immigr. Minor Health.

[12] Pérez-Escamilla R., Putnik P. (2007). The Role of Acculturation in Nutrition, Lifestyle, and Incidence of Type 2 Diabetes Among Latinos. J. Nutr..

[13] Darmon N., Drewnowski A. (2008). Does Social Class Predict Diet Quality?. Am. J. Clin. Nutr..

[14] Mozaffarian D. (2016). Dietary and Policy Priorities for Cardiovascular Disease, Diabetes, and Obesity: A Comprehensive Review. Circulation.

[15] Bhattacharya J., Currie J., Haider S. (2004). Poverty, food insecurity, and nutritional outcomes in children and adults. J. Health Econ..

[16] Leung C.W., Epel E.S., Ritchie L.D., Crawford P.B., Laraia B.A. (2014). Food insecurity and diet quality of lower-income adults. J. Acad. Nutr. Diet..

[17] Larson N., Laska M.N., Neumark-Sztainer D. (2020). Food insecurity and diet quality among emerging adults. Am. J. Public Health.

[18] Malik V.S., Pan A., Willett W.C., Hu F.B. (2013). Sugar-sweetened beverages and weight gain in children and adults. Am. J. Clin. Nutr..

[19] Khandelwal P., Salazar L.R. (2019). Exploring the social determinants of drinking sugary beverages leading to chronic illness among Latina/o populations. Hisp. Health Care Int..

[20] VanderWeele T.J., Knol M.J. (2014). A tutorial on interaction. Epidemiol. Methods.

[21] Florez K.R., Katic B.J., Lopez-Cevallos D.F., Murillo R., Cancel-Tirado D., Aponte-Soto L. (2019). The double burden of food insecurity and obesity among Latino youth. Pediatr Obes..

[22] Kuo S.C., Lee C.T., Chan K.C. (2023). Sugar-Sweetened Beverage Consumption and Metabolic Risks. Public Health Nutr..

[23] Barrett S.E., Gentry R., Pearson K.J. (2018). Beverage Preferences and Metabolic Risk. Public Health Nutr..

[24] Keller J.R., Hamer M., Goldsmith R. (2014). Gender Differences in the Association of SSB Consumption with Cardiovascular Health. Public Health Nutr..

[25] O’Mara J., Waterlander W., Nicolaou M. (2021). Exploring the Role of the Food Environment in Dietary Acculturation. Int. J. Environ. Res. Public Health.

[26] Dixon L.B., Sundquist J., Winkleby M. (2000). Differences in energy, nutrient, and food intakes in a US sample of Mexican-American women and men (NHANES III). Public Health Nutr..

[27] Mainous A.G., Diaz V.A., Geesey M.E. (2008). Acculturation and healthy lifestyle among Latinos with diabetes. Ann. Fam. Med..

[28] Maxwell S.L., Price J.C., Perito E.R., Rosenthal P., Wojcicki J.M. (2024). Food insecurity is a risk factor for metabolic dysfunction-associated steatotic liver disease in Latinx children. Pediatr. Obes..

[29] Ayala G.X., Baquero B., Klinger S. (2008). Acculturation and diet among Latinos in the United States: Implications. J. Am. Diet. Assoc..

[30] Cuy Castellanos D. (2015). Dietary acculturation in Latinos/Hispanics in the United States. Am. J. Lifestyle Med..

[31] Leider J., Powell L.M. (2019). Sugar-sweetened beverage prices: Variations by beverage, food store, and neighborhood characteristics, 2017. Prev. Med. Rep..

[32] Eaton T.M., Kumanyika S., DiSantis K.I., Yadeta K., Grier S. (2022). Black community conversations about opposing ethnically targeted marketing of unhealthy foods and beverages. J. Racial Ethn. Health Disparities.

[33] Bittman M. (2021). Animal, Vegetable, Junk: A History of Food, from Sustainable to Suicidal.

[34] Fielding-Singh P. (2021). How the Other Half Eats: The Untold Story of Food and Inequality in America.

[35] Ramírez A.S., Golash-Boza T., Unger J.B., Baezconde-Garbanati L. (2018). Questioning the Dietary Acculturation Paradox. J. Acad. Nutr. Diet..

[36] Flórez K.R., Bell B.M., Gálvez A., Hernández M., Verdaguer S., de la Haye K. (2023). Unraveling negative dietary acculturation among Mexican Americans in NYC. Appetite.

[37] Oza-Frank R., Cunningham S.A. (2010). The weight of US residence among immigrants: A systematic review. Obes. Rev..

[38] Robaina K.A., Martin K.S. (2013). Food insecurity, poor diet quality, and obesity among food pantry participants in Hartford. J. Nutr. Educ. Behav..

